# Supplementary material for: Exciton diffusion in two-dimensional metal-halide perovskites
Source: Nat Commun. 2020 Apr 27;11:2035. doi: 10.1038/s41467-020-15882-w (PMC7184754; doi:10.1038/s41467-020-15882-w)
Supplement: Supplementary file 1 — Supplementary Information [file 41467_2020_15882_MOESM1_ESM.pdf]

Supporting information

Exciton diffusion in two-dimensional metal-halide perovskites

Seitz et al.

Supplementary Figures

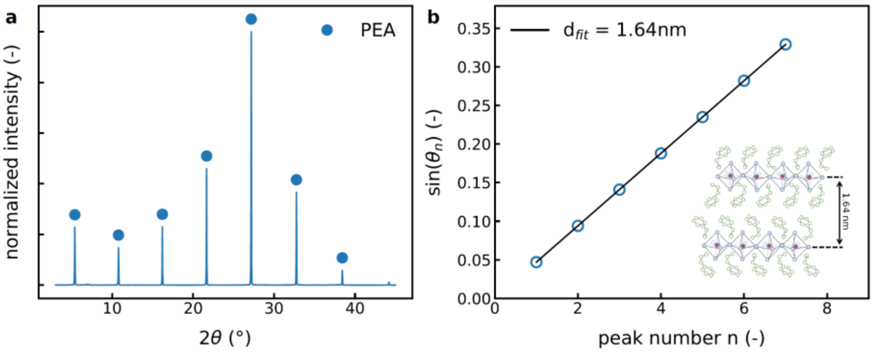

Supplementary Figure 1. a) XRD pattern of $(PEA)_2PbI_4$ b) In accordance with Bragg's law $\sin(\theta_n)$, where θ_n are the diffraction peak angles, follows a linear behavior and reveals a spacing of 1.64 nm between inorganic layers.

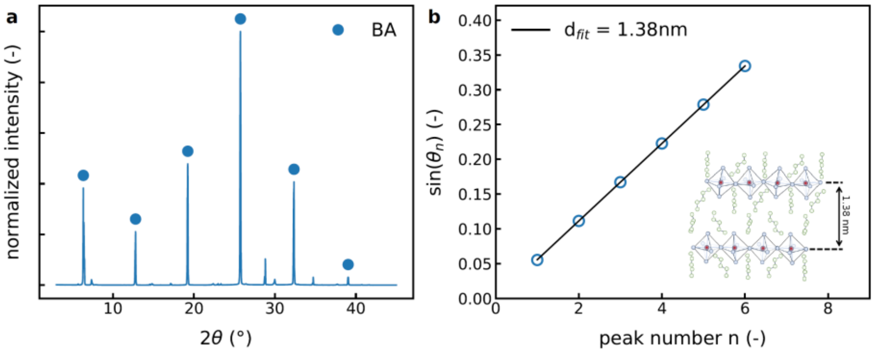

Supplementary Figure 2. a) XRD pattern of $(BA)_2PbI_4$ b) In accordance with Bragg's law $\sin(\theta_n)$, where θ_n are the diffraction peak angles, follows a linear behavior and reveals a spacing of 1.38 nm between the inorganic layers.

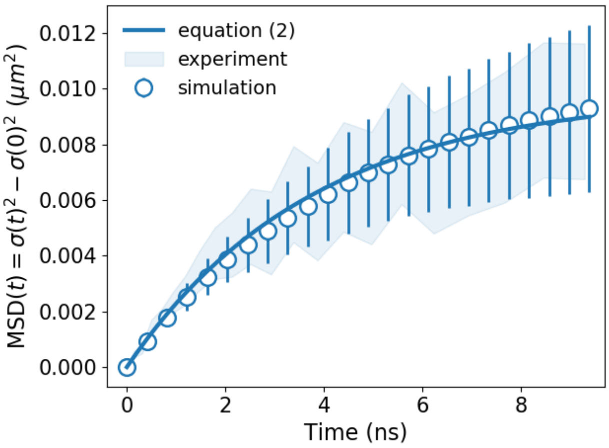

Supplementary Figure 3. Exciton diffusion in $(BA)_2PbI_4$. Experimental values (shaded area), theoretical model (Equation (2) of the main text, solid line), and numerical simulation (open markers). Experimental data is the same as in Fig. 3b in the main text. From Brownian motion simulations, we extract a trap density of around $1/\lambda^2 = 204\ \mu m^{-2}$. Reported errors represent the standard deviation of 10^4 Brownian motion simulations.

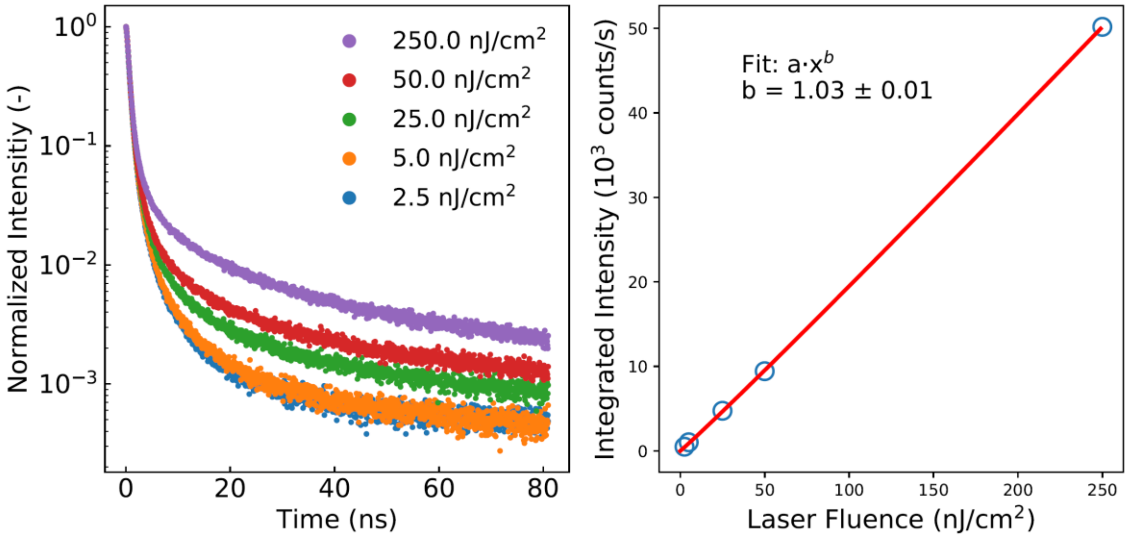

Supplementary Figure 4. a) Fluorescence lifetime traces of $(PEA)_2PbI_4$ for different laser fluences. b) Fluorescence intensity (integrated lifetime traces from a)) as a function of laser fluence.

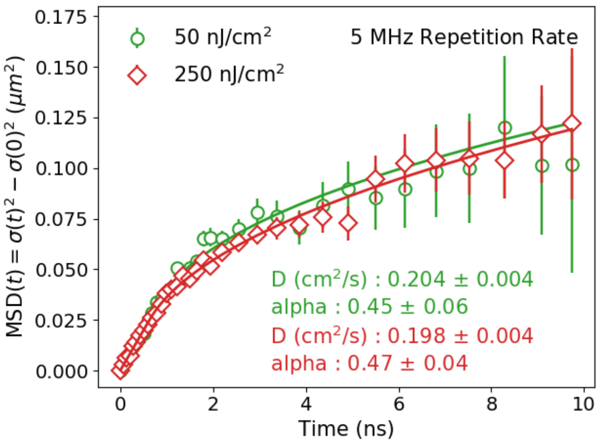

Supplementary Figure 5. MSD(t) from diffusion measurements with a repetition rate of 5 MHz and a laser fluence of 50 (open circles) and 250 nJ cm^{-2} (open diamonds), respectively. Reported errors represent the uncertainty in the fitting procedure for $\sigma(t)^2$.

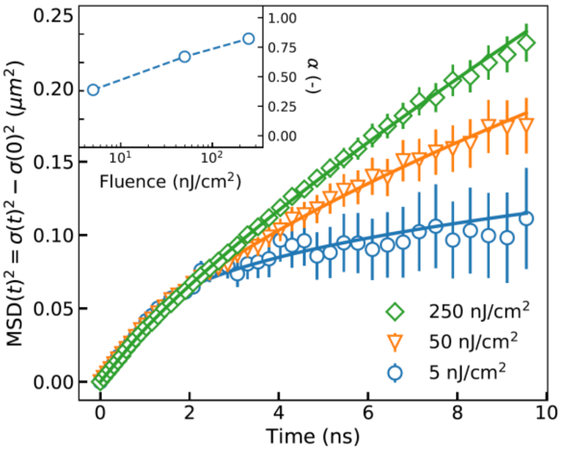

Supplementary Figure 6. Diffusion measurement for different laser fluences with a 40 MHz repetition rate. Higher laser fluences lead to an increased diffusion exponent α (inset), while the intrinsic diffusivity D stays unaffected (slope at early times). Reported errors represent the uncertainty in the fitting procedure for $\sigma(t)^2$.

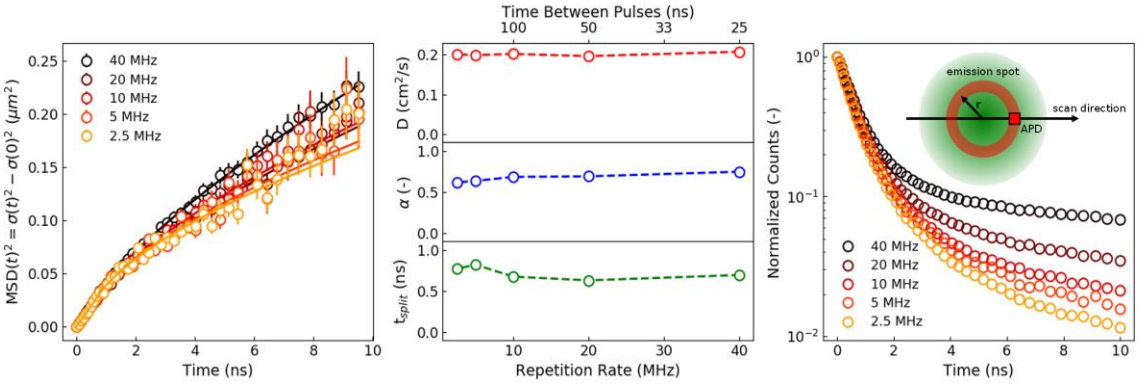

Supplementary Figure 7. a) MSD(t) from diffusion measurements with a 50 nJ cm^{-2} fluence and different laser repetition rates. Reported errors represent the uncertainty in the fitting procedure for $\sigma(t)^2$. b) Fit parameters from a): Diffusivity D, diffusion exponent α, and split of normal to subdiffusive regime t_{split}. c) Reconstructed lifetime trace from the diffusion data. The inset shows how the different lifetime traces of the diffusion measurement were weighted before summing them up to reconstruct the total fluorescence emission from the laser spot.

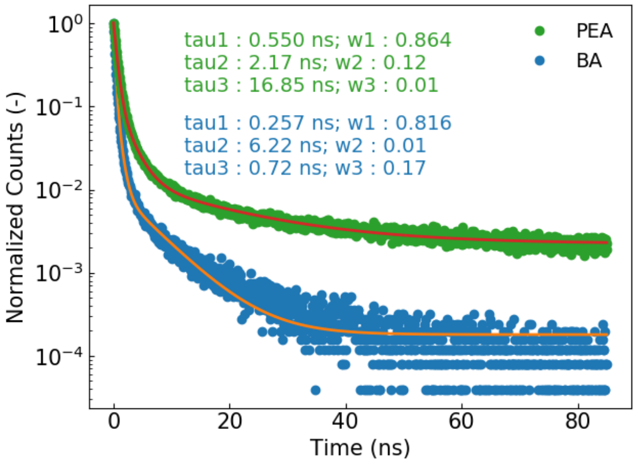

Supplementary Figure 8. Photoluminescence lifetime traces of $(PEA)_2PbI_4$ and $(BA)_2PbI_4$ with a tri-exponential fit. Fitting parameters are given in the figure.

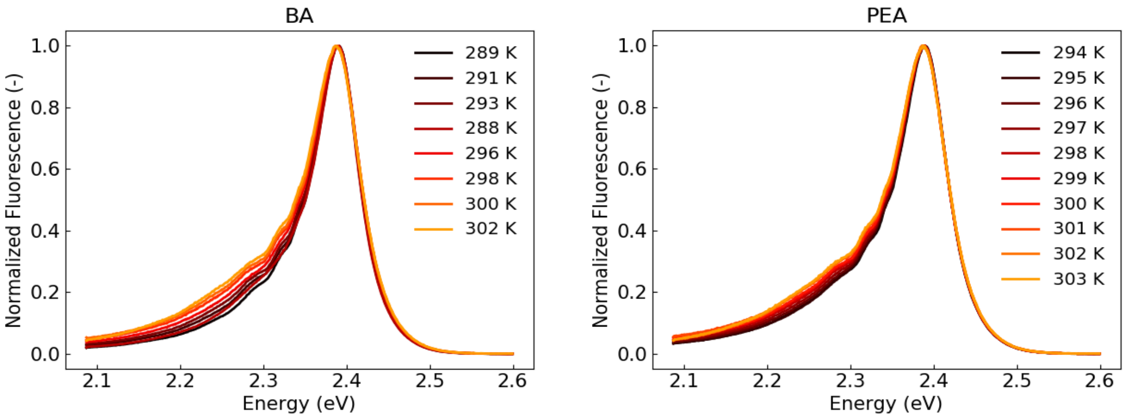

Supplementary Figure 9. Photoluminescence spectra of $(BA)_2PbI_4$ and $(PEA)_2PbI_4$ for different temperatures.

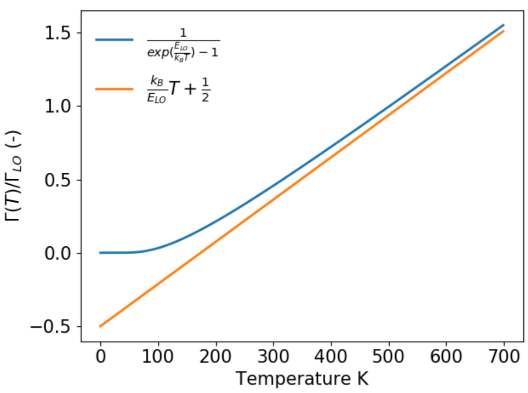

Supplementary Figure 10. Comparison of Supplementary Equation 24 and its asymptote Supplementary Equation 25 for $\Gamma_0 = 0$ and $E_{\mathrm{LO}} = 30$ meV.

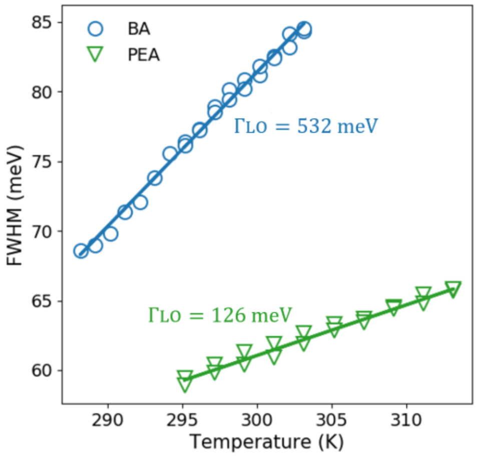

Supplementary Figure 11. Temperature dependent full-width-half-max $\Gamma(\mathrm{T})$ of the photoluminescence of $(PEA)_2PbI_4$ and $(BA)_2PbI_4$. and fits with Supplementary Equation 25.

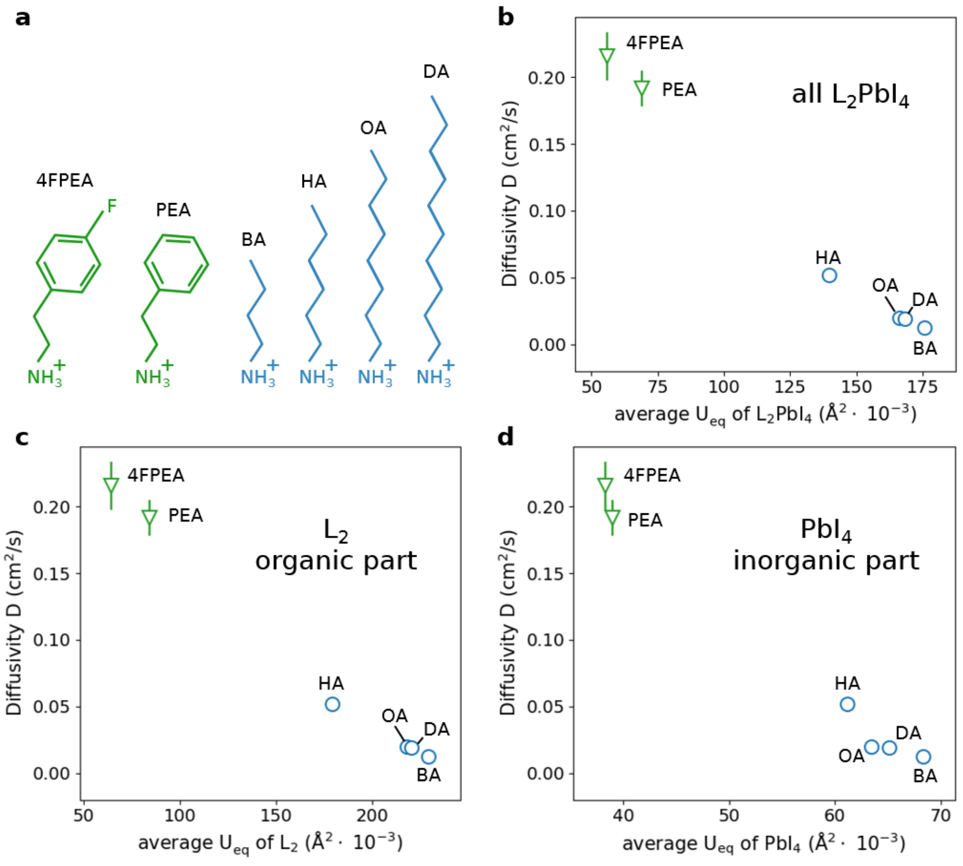

Supplementary Figure 12. a) Different organic spacers L used in this study (from left to right): 4-fluoro-phenethylammonium (4FPEA), phenethylammonium (PEA), butylammonium (BA), hexylammonium (HA), octylammonium (OA), decylammonium (DA). b) Diffusivity D vs. average atomic displacement U_{eq} of all the atoms in L_2PbI_4 perovskite (average $U_{eq} = \frac{1}{\#\,\text{atoms}} \sum_{\text{i=atom}}^{L_2PbI_4} U_{eq}^{i}$). c). D vs. average U_{eq} of the inorganic part L (average $U_{eq} = \frac{1}{\#\,\text{atoms}} \sum_{\text{i=atom}}^{L_2} U_{eq}^{i}$). d) D vs. average U_{eq} of the inorganic layer PbI_4 (average $U_{eq} = \frac{1}{5} U_{eq}^{Pb} + \frac{4}{5} U_{eq}^{I}$). Reported errors represent the standard deviation of the average diffusivity D obtained from multiple single crystalline flakes. U_{eq} data was extracted from previously published single crystal X-ray diffraction data.[1,2]

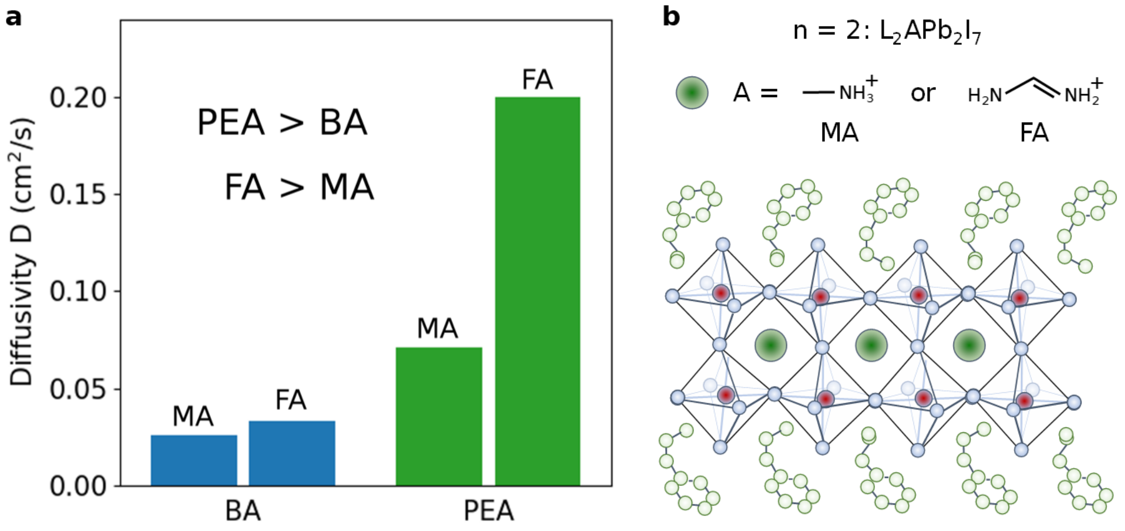

Supplementary Figure 13. a) Diffusivity for *n* = 2 perovskites, containing two octahedra per inorganic layer, with chemical formula $L_2APb_2I_7$. Organic spacers L were PEA or BA. Cation A were methylammonium (MA) or formamidinium (FA). Perovskites with PEA show higher diffusivities than perovskites with BA. Additionally, the diffusivity is higher if FA is used as cation instead of MA. b) Illustration of a single *n* = 2 perovskite ($L_2APb_2I_7$) layer.

Supplementary Tables

	d (nm)	d_{lit} (nm)	organic spacer L	cation A
**$(PEA)_2PbI_4$**	1.64	1.65[1]	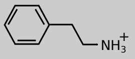	-
**$(4FPEA)_2PbI_4$**	1.66	1.67[3]	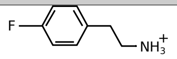	-
**$(BA)_2PbI_4$**	1.38	1.38[2]	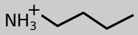	-
**$(HA)_2PbI_4$**	1.64	1.64[2]	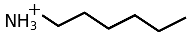	-
**$(OA)_2PbI_4$**	1.88	1.87[4]	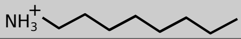	-
**$(DA)_2PbI_4$**	2.13	2.13[4]	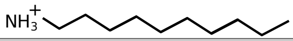	-
**$(BA)_2(MA)Pb_2I_7$**	1.97	1.96[5]	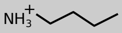	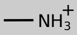
**$(BA)_2(FA)Pb_2I_7$**	1.97	1.96[5]	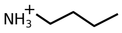	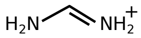

Supplementary Table 1. Summary of XRD measurements for perovskites in this paper. Table shows the lattice spacing d obtained in this study, reported lattice spacing d_{lit} from literature, and chemical drawing of the used organic spacer L and cation A of the perovskite ($L_2[APbI_3]_{n-1}PbI_4$).

	Γ_{LO} (meV)	E_{LO} (meV)
**$(PEA)_2PbI_4$**	126	41
**$(BA)_2PbI_4$**	532	30

Supplementary Table 2. Optical phonon coupling strength Γ_{LO} and optical phonon energy E_{LO} extracted from temperature dependent photoluminescence data of $(PEA)_2PbI_4$ and $(BA)_2PbI_4$.

Supplementary Note 1

Derivation of the mean-square-displacement (MSD) from the diffusion equation. For the theoretical model we followed the derivation by Akselrod et al.[6] For convenience we provide a summary here. The temporal and spatial exciton dynamics can be described by the general diffusion equation:

$$\dot{n}(x,t) = g(x,t) - \frac{n(x,t)}{\tau_{\mathrm{r}}} - \frac{n(x,t)}{\tau_{\mathrm{nr}}} + D\nabla^2 n(x,t), \tag{1}$$

where, $n(x,t)$ is the exciton density distribution, $g(x,t)$ the exciton generation term, τ_{r} the lifetime of radiative decay and τ_{nr} of non-radiative decay, D the diffusion constant and ∇ the gradient. Please note that higher order processes, such as exciton-exciton annihilation, are not taken into account because only low laser fluences are used in this study. The generation term can be assumed to be 0 for times larger than 0: $g(t > 0) = 0$ and can be accounted for in the initial conditions as $n(x,0) = g(x)$. In addition, the diffusion equation can be separated into a single dimension, because diffusion in x- and y-direction are uncorrelated, and our measurement takes a slice along a single Cartesian direction. For the sake of simplicity the radiative and non-radiative decay constants can be summarized in a single term as $\tau = \left(\frac{1}{\tau_{\mathrm{r}}} + \frac{1}{\tau_{\mathrm{nr}}}\right)^{-1} = \frac{\tau_{\mathrm{r}}\tau_{\mathrm{nr}}}{\tau_{\mathrm{r}}+\tau_{\mathrm{nr}}}$, without any loss of generality. The simplified one-dimensional diffusion equation then becomes

$$\dot{n}(x,t) = -\frac{n(x,t)}{\tau} + D\frac{\mathrm{d}^2 n(x,t)}{\mathrm{d}x^2}, \tag{2}$$

where $n(x,t)$ is the exciton density distribution, τ is the lifetime of the excitons and D is the diffusivity. The general solution with constant diffusivity D, constant decay rate τ, and an initial exciton distribution $n(x,0)$ is:

$$n(x,t) = \mathrm{e}^{-\frac{t}{\tau}} \frac{1}{\sqrt{4\pi Dt}} \int_{-\infty}^{\infty} n(x_0,0) e^{\frac{-(x-x_0)^2}{4Dt}} \mathrm{d}x_0 = \mathrm{e}^{-\frac{t}{\tau}}[n(x,0) * G(x,t)], \tag{3}$$

where $G(x,t) = \frac{1}{\sqrt{4\pi Dt}} \mathrm{e}^{-\frac{x^2}{4Dt}}$. This means that the exciton density distribution is a convolution of the initial exciton distribution $n(x,0)$ and a Gaussian function $G(x,t)$, with a variance of $\sigma(\mathrm{t})^2 = 2Dt$, multiplied with the temporal decay $\mathrm{e}^{-\frac{t}{\tau}}$ of the population. From Supplementary Equation 3 one can see that the lifetime τ of the excitons does not influence the broadening of the exciton population, allowing us to continue with the normalized quantity $\hat{n}(x,t) = \frac{\mathrm{n(x,t)}}{\mathrm{e}^{-\frac{t}{\tau}}}$. To approximate the initial exciton density distribution $n(x,0)$ a Lorentzian function is used, as it best describes the observed initial exciton distribution:[6]

$$\hat{n}(x,t) = n(\mathrm{x},0) * G(x,t) = L(x) * G(x,t) \tag{4}$$

In the experiment, the measured emission pattern $I(x,t)$ is a spatial convolution of the exciton distribution $\hat{n}(x,t)$, the APD detector $f_{\mathrm{d}}(x)$ and the point spread function (PSF) $f_{\mathrm{psf}}(x)$ of the optical system:

$$I(x,t) = \hat{n}(x,t) * f_{\mathrm{d}}(x) * f_{\mathrm{psf}}(x) = L(x) * G(x,t) * f_{\mathrm{d}}(x) * f_{\mathrm{PSF}}(x) \tag{5}$$

The APD detector size of 20 µm, which when imaged onto the sample with a 330x magnification becomes 60 nm, is small when compared to the laser spot size. Therefore, the influence of the APD detector size is negligible and Supplementary Equation 5 can be rewritten as

$$I(x,t) = L(x) * G(x,t) * f_{\mathrm{PSF}}(x) \tag{6}$$

Approximating the PSF of the microscope with a Gaussian function and knowing that a convolution of two Gaussians is a gaussian as well, allows us to further simplify the equation:

$$I(x,t) = L(x) * G(x,t) * f_{\mathrm{PSF}}(x) = L(x) * \tilde{G}(x,t) \propto L(x) * \mathrm{e}^{-\frac{x^2}{2\sigma_I^2(t)}} \tag{7}$$

Since the variance of convoluted Gaussians is additive we can write $\sigma_I^2(\mathrm{t}) = 2Dt + \sigma_{\mathrm{PSF}}^2$, where $\sigma_I^2(t)$ is the variance of $\tilde{G}(x,t)$, $2Dt$ is the variance due to the diffusion term $G(x,t)$, and σ_{PSF}^2 is the variance of the point spread function of the microscope, which is constant over time.

Fitting the experimental data $I(x,t)$ with a Voigt function (convolution of Lorentzian and Gaussian function), allows the extraction of the full-width-half-maximum (fwhm) of the Lorentzian part $L(x)$ and the variance $\sigma_I^2(\mathrm{t})$ of the Gaussian part $\tilde{G}(x,t)$. As a result, the mean-square-displacement (MSD) of an exciton can be calculated by subtracting the variance of the Gaussian ($\sigma_I^2(t)$) by the variance at time 0 ($\sigma_{\mathrm{I}}^2(0)$):

$$MSD(t) = \sigma_I^2(t) - \sigma_I^2(0) = 2Dt + \sigma_{\mathrm{PSF}}^2 - \sigma_{\mathrm{PSF}}^2 = 2Dt \tag{8}$$

Consequently, the diffusivity D can be extracted by measuring the dime-dependent variance $\sigma_{\mathrm{I}}^2(t)$ of the exciton distribution.

To extract the MSD of the exciton population for different times t, we fit the population with a Voigt distribution. A Voigt function is completely defined through five parameters: The fwhm of the Lorentzian part Γ, the variance of the Gaussian part σ^2, the center of the distribution μ, the proportionality factor A, and the offset c due to, for example, background noise: $V(x) = A\left(\frac{\Gamma/2}{(x-\mu)^2+\Gamma^2/4} * \mathrm{e}^{-\frac{(x-\mu)^2}{2\sigma^2}}\right) + c$. Γ, μ and c are constant for all times, while σ and A change over time. In our fitting procedure we fit the data such that for every time-slice we allowed different σ_k and A_k parameters (indexed here with k), while Γ, μ and c were shared and constant for all times. As a result, the fitting includes 3 + 2k parameters. The fitting is performed

in python with the optimize.curve_fit function of the scipy package. Although we are only interested in the width of the exciton population (σ_k), we did fit the non-normalized data as this guarantees that every data point of $I(x,t)$ is weighted according to its signal-to-noise (S/N) ratio. If the normalized data would be used, data points for late times would get weighted the same as early time points in the fitting procedure, despite a much inferior S/N ratio of data points collected at later times.

The S/N of fluorescence lifetime data significantly decreases for later times due to the exponential behavior. As a result, we choose to apply a non-linear binning to our data before the fitting procedure. Meaning at early times fewer bins are added together ($I_{\mathrm{binned}} = \sum_{k=i}^{i+n_{early}} \frac{I_k}{n_{\mathrm{early}}}$) than for later times ($I_{binned} = \sum_{k=i}^{i+n_{\mathrm{late}}} \frac{I_k}{n_{\mathrm{late}}}$). Hence, $n_{\mathrm{early}} < n_{\mathrm{late}}$. The number of bins is chosen according to the following function: $n_k = \mathrm{int}(0.3k^{1.5} + 16) \rightarrow [16, 16, 16, 17, 18, 19, 20, 21, 22, 24 \ldots]$. The time bin size before binning was 4 ps.

Binning improves the S/N ration of the data. With non-linear binning, the $(\mathrm{S/N})_k$ for every time-slice improves differently. As a result, every time-slice is weighted with the number of bins n_k to correctly account for the improved $(\mathrm{S/N})_k$ due to binning. This multiplication with n_k has an influence on the actual offset parameter *c*, that needs to be fitted to each time-slice k. After non-linear binning, every time-slice needs to be fitted with a local offset of $c_k = c \cdot n_k$, with c being the real/global offset of the data. Following this procedure, we are able to extract the variance σ_k^2 ($\sigma_I^2(t)$ in the previous section) for every time-slice, which are then used to generate the MSD ($= \sigma_I^2(t) - \sigma_I^2(0) = \sigma_k^2 - \sigma_0^2$) vs. time plots, that allowed the extraction of the diffusion parameters *D*, α and t_{split}. Fig. 1d shows a subset of the fitted Voigt functions together with the experimental data, which were normalized to 1 for a better comparison. Please note that the real offset for all curves is the same. The apparent increase of the offset for later times is solely due to the normalization of the data.

We would like to note that the shortest lifetime of some perovskites is close to the width of the pulse-width of the pulsed laser diode. To minimize the influence of the laser and justify the assumption of negligible exciton generation during our measurements ($g(t > 0) = 0$) we only analyze the diffusion data 250 ps after the maximum of the photoluminescence lifetime data and define it as the new t = 0.

In the previous paragraphs, we derived that the MSD of normal diffusion in one-dimension to be: $\mathrm{MSD}(t) = 2Dt$. However, diffusion in disordered media, where the diffusivity is not constant for all times, is better described by introducing the diffusion exponent α: $\mathrm{MSD}(t) = 2Dt^{\alpha}$.[6–8] With the diffusion exponent α one can describe normal diffusion ($\alpha = 1$),

superdiffusion ($\alpha > 1$, e.g. ballistic transport) and subdiffusion ($\alpha < 1$, e.g. through trapping of excitons).

As described in the main text the MSD(t) of excitons in 2D perovskites shows two distinct diffusion regimes: First, a linear behavior of normal diffusion ($2Dt^{\alpha},\ \alpha = 1$), which is followed by a second subdiffusive regime ($2Dt^{\alpha},\ \alpha < 1$). We fit the two regimes simultaneously with the following fit function:

$$\mathrm{MSD}(t) = \begin{cases} 2Dt + c & for\quad t \leq t_{\mathrm{split}} \\ 2D\left[\left(t - t_{\mathrm{split}} + t_0\right)^{\alpha} + t_{\mathrm{split}} - t_0^{\alpha}\right] + c & for\quad t > t_{\mathrm{split}} \end{cases} \tag{9}$$

with the fit parameters *D*, α, *c*, and t_{split}. c is generally small and is introduced to avoid overweighing the first datapoint at time t = 0. $t_0 = \alpha^{\frac{1}{1-\alpha}}$ was introduced to make MSD(t) continuous in value (excitons move continuously) and slope (speed of excitons changes continuously) at time t_{split}. By fitting our experimental data with Supplementary Equation 9 we were able to extract the diffusivity *D*, diffusion exponent α, and the onset of subdiffusion t_{split} from our measurements. We would like to note that fitting only the first linear regime of normal diffusion with $2Dt^{\alpha}$ yields almost identical *D* values and α values of around 1.

Supplementary Note 2

Influence of laser fluence. Supplementary Figure 4 shows the lifetime traces of $(PEA)_2PbI_4$ recorded with a 10 MHz repetition rate and for different laser fluences of a near-diffraction limited spot. All traces show a multiexponential decay and the same early time dynamics, which shows that exciton-exciton annihilation is absent for laser fluences used in this study. On the other hand, the slow decaying component becomes slightly (logarithmic y-axis) more prominent with increasing laser fluence, which could be due to trap state filling. This is further supported by Supplementary Figure 4b, where the total emission intensity (integrated lifetime data) is shown as a function of laser fluence. Exciton-exciton annihilation would result in sublinear behavior, but instead a slightly superlinear behavior is observed, which could originate from trap state filling.

Influence of repetition rate. The influence or trap state filling is absent in the diffusion measurements performed with laser repetition rates of 5 MHz as shown in Supplementary Figure 5. For both 50 $nJcm^{-2}$ and 250 $nJcm^{-2}$, the diffusivity and diffusion exponent are the same.

However, for diffusion measurements performed with a repetition rate of 40 MHz (standard repetition rate of this report) the diffusion exponent increases with increasing laser fluences, while the diffusivity stays constant. An increased diffusion exponent indicates a lower

effective trap density, which is likely due to trap state filling. As a result, the intrinsic diffusivity *D* can be extracted for any laser repetition rate, while it is necessary to use low repetition rates to measure the real trap state density of the perovskites.

The finding that the laser fluence only influences the diffusion measurements for high repetition rates (40 MHz, Supplementary Figure 6), but not for low ones (5 MHz, Supplementary Figure 5), suggests that the traps are filled with excitons from previous pulses rather than from excitons which are generated with the same laser pulse. This can be explained by a long lifetime of trap states. With increasing laser fluence a higher percentage of laser pulses create excitons in the same inorganic layer as the following laser pulse, allowing the first exciton to fill a trap and thereby reducing the effective trap density experience by a second exciton generated by a later laser pulse.

For a better understanding of the trap state filling, we performed diffusion measurements with different repetition rates and a constant fluence of 50 $nJ\,cm^{-2}$ (Supplementary Figure 7). All measurements show the same diffusivity and comparable, but slightly increasing diffusion exponents α (Supplementary Figure 7b). It is possible to reconstruct the standard lifetime trace of the whole laser spot, which does not show any diffusion dynamics, by summing up all the lifetime traces of a diffusion experiment (traces at all positions). However, before the summation, the individual lifetime traces (from different scanning positions) need to be weighted with a factor of $2\pi r$ to account for the whole 2D emission spot, with r being the distance of the APD from the center of the laser spot (see inset in Supplementary Figure 7c). The resulting lifetime traces are shown in Supplementary Figure 7c. The lifetime traces acquired with a repetition rate of 2.5, 5, and 10 MHz are almost identical, suggesting a trap state lifetime on the order of 100 ns (= 1/10 MHz).

To guarantee a good signal to noise ratio most measurements in this study were performed with a 40 MHz laser repetition rate. As outlined above, this only changes the observed effective trap density (and thereby diffusion exponent α) but does not influence the intrinsic diffusivity of the material.

Supplementary Note 3

Brownian modelling of trap states. We have performed Brownian dynamics simulations of a single exciton diffusing in a field of traps, representing ideal (non-interacting) excitons in the dilute limit carried out in experiments. In these simulations, an exciton diffuses freely until it finds a trap, where it just stops. Free diffusion is modelled using the standard stochastic

differential equation for Brownian motion in the Itô interpretation. If $\mathbf{r}(t)$ is the position of the exciton in the plane at time t, its displacement $\Delta\mathbf{r}$ over a time Δt is given by,

$$\Delta \boldsymbol{r} = \sqrt{2D}\,\mathrm{d}\mathbf{W}, \tag{10}$$

where D is the free-diffusion coefficient and d$\mathbf{W}$ is taken from a Wiener process, such that $\langle \mathrm{d}\mathbf{W}\mathrm{d}\mathbf{W}\rangle = \Delta t$. Traps were scattered throughout the plane following a uniform random distribution. The exciton is considered to be trapped as soon its location gets closer than R_{trap} = 1.2 nm to the trap center. The value was taken from estimations of the exciton Bohr radius and corresponds to a trap area of 1.44 nm^2.[9] In any case, in the dilute regime, the diffusion is not sensitive to the trap size R_{trap}, because the trap radius is much smaller than the average separation between traps, $R_{\mathrm{trap}} \ll \lambda$. To numerically integrate the equation of motion, we used a simple second-order-accurate modification of the well-known Euler Maruyama algorithm: the BAOAB-Limit method.[10] Trajectories were computed for many independent excitons and the data was averaged to determine the MSD as a function of time. While the simulation of the MSD was done in two dimensions, we used the MSD in one dimension to match the experimental conditions: $\mathrm{MSD}(t) = \frac{1}{2}\left(\mathrm{MSD_x}(t) + \mathrm{MSD_y}(t)\right)$.

Supplementary Note 4

Derivation of continuum model. Besides studying the exciton dynamics using Brownian dynamics simulations, we derive a continuum model for the exciton diffusion in a plane having a uniform random distribution of traps. This coarser model solves the equation for the field of exciton concentration, or equivalently, for the probability field $c(\mathbf{r},t)$ of finding an exciton at location $\mathbf{r}$ at time t. The resulting partial differential equation for $c(\mathbf{r},t)$ is numerically solved using a finite difference scheme in a rectangular mesh of size h. Thus, in this description, $\boldsymbol{r}$ corresponds to the center of a given control cell of the mesh, whose area h^2 is much larger than the trap area $s_0 = \pi R_{\mathrm{trap}}^2$, but yet much smaller than the system size L. In other words, $R_{\mathrm{trap}} \ll h \ll L$. To model the spatial distribution (the MSD), we consider two kinds of excitons: mobile excitons which freely diffuse with a diffusion constant D and trapped excitons which stand still. Note that the temporal dynamics, such as the radiative lifetime of excitons, can be neglected since we are only interested in the spatial dynamics. The total concentration of excitons is just

$$C_{\mathrm{tot}}(\mathbf{r},t) = c(\mathbf{r},t) + c_{\mathrm{t}}(\mathbf{r},t), \tag{11}$$

where $c(\mathbf{r},t)$ is the concentration of mobile excitons and $c_{\mathrm{t}}(\boldsymbol{r},t)$ that of the trapped ones. Any increase in trapped excitons is due to a loss in mobile excitons, $\delta c_{\mathrm{t}}(\mathbf{r},t) = -\delta c(\mathbf{r},t)$. The

concentration of mobile excitons will obey a reaction-diffusion equation, with a free diffusion term ($D\nabla^2 c$) and sink or loss rate term, *s(**r**,t)*,

$$\partial_{\mathrm{t}} c(\mathbf{r}, t) \ = \ D\nabla^2 c(\mathbf{r}, t) - s(\mathbf{r}, t). \tag{12}$$

The equation for trapped excitons is then simply, $\partial_{\mathrm{t}} c_{\mathrm{t}}(\mathbf{r}, t) \ = \ s(\mathbf{r}, t)$, and that for the total number of excitons,

$$\partial_{\mathrm{t}} C_{\mathrm{tot}}(\mathbf{r}, t) \ = \ \nabla \cdot \big(D\nabla c(\mathbf{r}, t)\big) \tag{13}$$

which is explicitly written in conservative form: note that the total exciton flux is just the diffusive flux of mobile excitons $\mathbf{j} = \ -D\nabla c$.

As we show below, the exciton's mean square displacement is directly given by Supplementary Equation 13. But, at this stage one needs to model the loss rate *s*(***r**,t*), provided that the surface fraction of trap p is fixed, and the trap area is s_0. Recall that the average distance between traps is λ so, $p = s_0/\lambda^2$ and the trap density is p/s_0. The loss rate has two contributions. First, over a time lapse Δ*t*, the excitons located at the cell ***r*** (whose number is *c*(***r**,t*) h^2) will diffuse an area $A = \gamma D_0 \Delta t$ (where γ represents a dimensionless proportionality factor), and a fraction $p\, A/s_0$ of them will fall in a trap. The second contribution to *s*(***r**,t*) is due to the flux of excitons across the boundaries of the cell ***r***. The number of excitons crossing the boundaries of the cell ***r*** (to or from another cell) is just

$$\int \mathbf{j} \cdot n \mathrm{d}\mathbf{r} = \int \nabla \cdot (D\nabla c) \mathrm{d}\mathbf{r}^2 \ \approx \ h^2 D \nabla^2 c. \tag{14}$$

Here, we have used the Gauss divergence theorem and the fact that the cell size h is infinitesimal. Again, a fraction p of these excitons will also fall in a trap. In summary, the sink (loss rate) is given by

$$s(\mathbf{r}, t) \ = \ \gamma p D \nabla^2 c(\mathbf{r}, t) \ + \ \gamma \frac{p}{s_0} D c(\mathbf{r}, t), \tag{15}$$

and the resulting equation for the mobile excitons is

$$\partial_{\mathrm{t}} c(\mathbf{r}, t) \ = \ D(1 - \gamma p)\nabla^2 c(\mathbf{r}, t) \ - \ \gamma p \frac{D}{s_0} c(\mathbf{r}, t). \tag{16}$$

This equation has a particularly simple analytical solution in Fourier space,

$$\partial_{\mathrm{t}} \tilde{c}(\mathbf{q}, t) \ = \ -D(1 - \gamma p)\mathbf{q}^2 \tilde{c}(\mathbf{q}, t) \ - \ \gamma \frac{D_0}{\lambda^2} \tilde{c}(\mathbf{q}, t), \tag{17}$$

with $\tilde{c}(\mathbf{q}, t) \ = \ \tilde{c}(t) \mathrm{e}^{-\mathrm{i}\mathbf{q}\cdot\mathbf{r}}$. The solution of Supplementary Equation 17 is simply:

$$\tilde{c}(\mathbf{q}, t) \ = \ \exp\{-D(1 - \gamma p)\mathbf{q}^2 t\} \exp\left(-\gamma \frac{\mathrm{D}}{\lambda^2}\, t\right). \tag{18}$$

This expression for the concentration of free excitons in Fourier space will be used later to obtain an analytical expression for the diffusion coefficient.

It is now possible to obtain an exciton's mean-square-displacement MSD(t) = $\sigma^2(t)$ from the second moment of the time dependent probability distribution for the exciton location $C_{tot}(\boldsymbol{r},t)$ in Supplementary Equation 13. Without loss of generality, we assume that the average position of the excitons is centered at zero **r** = *0* (i.e. $\int \mathbf{r}\, C_{\text{tot}}(\mathbf{r},t)\mathrm{d}\mathbf{r}^2 = 0$) so that, by definition,

$$\frac{d\sigma^2(t)}{dt} = \int \mathbf{r}^2\, [\partial_t C_{\text{tot}}(\mathbf{r},t)] d\boldsymbol{r}^2 = D \int \mathbf{r}^2\, [\nabla^2 c(\mathbf{r},t)]\mathrm{d}\mathbf{r}^2. \tag{19}$$

Integrating twice by parts and using natural boundary conditions (absorbing boundaries) for $C_{tot}(\mathbf{r},t)$,[11] one gets

$$\frac{\mathrm{d}\sigma^2(t)}{\mathrm{d}t} = 2D \int c\,(\mathbf{r},t)\mathrm{d}\mathbf{r}^2. \tag{20}$$

The time-dependent diffusion coefficient is just the derivative $D(t) = \frac{1}{2}\frac{\mathrm{d}\sigma^2(t)}{\mathrm{d}t}$. Thus, the combination of Supplementary Equations 18 and 20 leads to

$$\begin{aligned} D(t) &= D\int c\,(\mathbf{r},t)\mathrm{d}r^2 = D\int \mathrm{d}\mathbf{r}^2 \int \tilde{c}\,(\mathbf{q},t)\mathrm{e}^{\mathrm{i}\mathbf{q}\cdot\mathbf{r}}\mathrm{d}q = D\int \tilde{c}\,(\mathbf{q},t)\left[\iint \mathrm{e}^{\mathrm{i}\mathbf{q}\cdot\mathbf{r}}\,\mathrm{d}\mathbf{r}^2\right]\mathrm{d}\mathbf{q} \\ &= D\int \delta\,(\mathbf{q})\tilde{c}(\mathbf{q},t)\mathrm{d}\mathbf{q} = D\tilde{c}(0,t) = D\exp\left(-\gamma\frac{D}{\lambda^2}\,t\right) \end{aligned} \tag{21}$$

where in the last step we have used Supplementary Equation 18 above. Integrating Supplementary Equation 21 leads to the MSD plotted in Fig. 2c of the main text,

$$\mathrm{MSD}(t) \equiv \sigma^2(t) = 2\frac{\lambda^2}{\gamma}\left[1 - \exp\left(-\gamma\frac{D}{\lambda^2}\,t\right)\right]. \tag{22}$$

The derived exponential decay of the diffusion coefficient is found to be in good agreement with the experimental results with γ = 1 and permits the determination of the free diffusion coefficient D and the trap density $1/\lambda^2$. While the derivation of the MSD was done in two dimensions, we used the MSD in one dimension to match the experimental conditions: $\mathrm{MSD}(t) = \frac{1}{2}\left(\mathrm{MSD_x}(t) + \mathrm{MSD_y}(t)\right)$.

Calculation of sun-equivalent. The calculation of the sun-equivalent in Fig. 2c was performed with the AM1.5 Global (ASTMG173) standard spectra. We extract that 1 sun contains $4.8 \cdot 10^{16}$ photons s^{-1} cm^{-2} with an energy larger than the bandgap of $(PEA)_2PbI_4$ ($\lambda_{\text{photon}} < \lambda_{\text{bandgap}} \approx 520$ nm). Assuming that the absorption of a photon with above-bandgap energy is wavelength independent and that every absorbed photon creates an exciton, one finds a 1 sun equivalent of 25 mW cm^{-2} for a 385 nm light source. In other words, in a first approximation, 1 sun should excite an equal number of excitons in $(PEA)_2PbI_4$ as the illumination with 25 mW cm^{-2} at 385 nm.

Evaluation of trap-density in $(BA)_2PbI_4$. We fit our continuum model (Equation 2) to our experimental data and performed numerical simulations based on Brownian dynamics for both $(PEA)_2PbI_4$ (see Fig. 2c) and $(BA)_2PbI_4$ (see Supplementary Figure 3). We find trap state densities

of 22 μm^{-2} and 204 μm^{-2} for $(PEA)_2PbI_4$ and $(BA)_2PbI_4$, respectively. We would like to emphasize that despite the difference in trap state density, a clear linear ($\alpha = 1$) behavior at early times can be identified for both $(PEA)_2PbI_4$ and $(BA)_2PbI_4$ (see Fig. 3b), consistent with unobstructed exciton diffusion (through e.g. traps) at early times, giving us access to the intrinsic diffusivity of these materials (see Fig. 2 for more information on the trap states on the MSD).

Supplementary Note 5

Determination of diffusion length. Fluorescence lifetime measurements were performed using a laser diode of λ = 405 nm (PicoQuant LDH-D-C-405, PDL 800-D, Pico-Harp 300) and an avalanche photodiode (APD, Micro Photon Devices PDM). The repetition rate was 10 MHz and the peak fluence per pulse was 50 nJ cm^{-2}. Supplementary Figure 8 shows the photoluminescence lifetime traces of $(PEA)_2PbI_4$ and $(BA)_2PbI_4$, and a tri-exponential fit to the data. The fit was used together with the experimentally obtained time-dependent MSD to extract the total number of surviving excitons for a given time t as presented in Fig. 3b of the main text. The total number of surviving excitons at time t is given by:

$$\text{surviving excitons (t)} = \frac{\int_0^t w_1 e^{-\frac{t}{\tau_1}} + w_2 e^{-\frac{t}{\tau_2}} + w_3 e^{-\frac{t}{\tau_3}}\, dt}{\int_0^\infty w_1 e^{-\frac{t}{\tau_1}} + w_2 e^{-\frac{t}{\tau_2}} + w_3 e^{-\frac{t}{\tau_3}}\, dt} \qquad (23)$$

Supplementary Note 6

Temperature dependent photoluminescence linewidth. Fluorescence spectra were measured using a spectrograph with a 300 g/mm grating with a blaze of 500 nm (SpectraPro HRS-300) and an EMCCD camera (ProEM HS 1024BX3) from Princeton Instruments. The perovskites were excited with a blue LED (Thorlabs M385PLP1-C5, λ = 385 nm). The temperature of the perovskite crystal was varied with a Peltier element (Adaptive Thermal Management, ET-127-10-13-H1), using a PID temperature controller (Dwyer Instruments, Series 16C-3) connected to a type K thermocouple (Labfacility, Z2-K-1M) for feedback control and a fan for cooling. Supplementary Figure 9 shows the temperature dependent emission spectrum for $(BA)_2PbI_4$ and $(PEA)_2PbI_4$. We applied the Jacobian conversion described by Mooney and Kambhampati to switch from wavelengths to energies.[12]

As several studies have shown before, the temperature dependent FWHM $\Gamma(T)$ of photoluminescence of bulk[13–16] and 2D[17–22] perovskites can be described with the Bose−Einstein distribution, due to thermal occupation of phonon modes:

$$\Gamma(T) = \frac{\Gamma_{\mathrm{LO}}}{\mathrm{e}^{\frac{E_{LO}}{k_B T}} - 1} + \Gamma_0 \tag{24}$$

where Γ_{LO} is the optical phonon coupling strength, E_{LO} is the optical phonon energy, k_{B} is the Boltzmann constant, and Γ_0 is the zero phonon linewidth.[23–25] For high temperatures, where the thermal energy is much greater than the phonon energy Supplementary Equation 24 can be approximated through its asymptote:

$$\Gamma(T) = \frac{\Gamma_{\mathrm{LO}}}{e^{\frac{E_{LO}}{k_B T}} - 1} + \Gamma_0 \xrightarrow{k_B T \gg E_{LO}} \frac{\Gamma_{\mathrm{LO}}}{E_{LO}} k_B T - \frac{\Gamma_{\mathrm{LO}}}{2} + \Gamma_0 \tag{25}$$

Literature values for $(PEA)_2PbI_4$ and $(BA)_2PbI_4$ range between around 10 to 20 meV for Γ_0 and 10 to 40 meV for E_{LO}. In Supplementary Figure 10, we plot Supplementary Equation 24 and its asymptote, Supplementary Equation 25, for $\Gamma_0 = 0$ and a phonon energy $\mathrm{E_{LO}} = 30meV$. One can see that Supplementary Equation 24 follows the linear behavior of its asymptote (Supplementary Equation 25) already for $T \gtrsim 300K$. As a result, we used Supplementary Equation 25 to fit our temperature dependent data and extract the optical phonon coupling strength Γ_{LO} and optical phonon energy E_{LO} for $(PEA)_2PbI_4$ and $(BA)_2PbI_4$ (see Supplementary Figure 11). The resulting Γ_{LO} and E_{LO} values are listed in Supplementary Table 2. $\mathit{\Gamma}_0$ was assumed to be 15 meV in accordance with literature values[17–22,26]. The exact value of $\mathit{\Gamma}_0$ is not critical for the fit, because $\mathit{\Gamma}_{\mathrm{LO}}$ is normally significantly greater than $\mathit{\Gamma}_0$. Our values, fit well with the previously reported values for $(PEA)_2PbI_4$[17–20,26] and $(BA)_2PbI_4$.[21,22] However, we would like to note that due to the uncertainties in this technique the values listed in Supplementary Table 2 should not be taken as quantitative results, but rather qualitative results to demonstrate the stronger exciton-phonon interaction in $(BA)_2PbI_4$ as compared to $(PEA)_2PbI_4$.

Supplementary Note 7

Diffusivities for different chemical compositions. Supplementary Figure 12 shows the diffusivity values for *n* = 1 perovskites (L_2PbI_4) with different organic spacers L. Plotting the diffusivity values as a function of average atomic displacement U_{eq} reveals an inverse relation and highlights the correlation of lattice softness and diffusivity. Supplementary Figure 12 shows the plots for average atomic displacements (average $U_{\mathrm{eq}} = \frac{1}{\#\,\mathrm{atoms}} \sum_i U_{\mathrm{eq}}^i$) of the whole perovskite L_2PbI_4 (panel b), as well as separated for the organic spacer molecule L (panel c), and the inorganic layer PbI_4 (panel d). U_{eq} values were extracted from literature from single crystal x-ray diffraction data: 4-fluoro-phenethylammonium (4FPEA),[3] phenethylammonium (PEA),[1] butylammonium (BA),[2] hexylammonium (HA),[2] octylammonium (OA),[4] decylammonium (DA).[4]

Supplementary Figure 13a shows how the diffusivity changes for n = 2 perovskites ($L_2[APbI_3]_{n-1}PbI_4$) for different organic spacers L (PEA and BA) and cations A (methylammonium (MA) and formamidinium (FA)). We find that the organic spacer PEA yields higher diffusivities than BA, just like in the n = 1 case. As for the cation, FA yields higher diffusivities than MA. FA is a larger molecule than MA and fills out the PbI_6-octahedra cage more completely. As a result, the PbI_6 octahedra are tilted less for FA than for MA. Higher tilt angles were found to yield higher effective electron and hole masses in perovskites.[27,28] In addition, FA being a bulkier molecule results in a more rigid crystal as supported by the average atomic displacement values of the inorganic part (average $U_{\mathrm{eq}} = \frac{2}{9} U_{\mathrm{eq}}^{\mathrm{Pb}} + \frac{7}{9} U_{\mathrm{eq}}^{\mathrm{I}}$) of $(BA)_2MAPb_2I_7$ and $(BA)_2FAPb_2I_7$ being 0.098 and 0.089 Å^2, respectively.[5]

It is important to note that Gélvez-Rueda et al. have measured an exciton to free carrier fraction of around 50 % for the n = 2 perovskite $(BA)_2FAPb_2I_7$, suggesting that transport is not purely excitonic.[29] As a result, our current results serve as a qualitative scaling of the diffusivity and a more rigorous analysis of the n = 2 data would be needed for quantitative diffusivity values.

Supplementary Methods

Chemicals. Chemicals were purchased from commercial suppliers and used as received: *MX_2:* lead(II) iodide (PbI_2) (Sigma Aldrich, 900168-5G).

LX: phenethylammonium iodide (PEAI) (Sigma Aldrich, 805904-25G), n-butylammonium iodide (BAI) (Sigma Aldrich, 805874-5G), n-octylammonium iodide (OAI) (Greatcell Solar Materials, MS105500-5), 4-fluoro-phenethylammonium iodide (4FPEAI) (Greatcell Solar Materials, MS100720-05)

AX: formamidinium iodide (Greatcell Solar Materials, MS150000-05), methylammonium iodide (Sigma Aldrich, 793493-5G).

Solvents: γ-butyrolactone (Sigma Aldrich, B103608-500G)

n-hexylammonium iodide (HAI) and n-decylammonium iodide (DAI) were not purchased directly from a commercial supplier but were synthesized by reacting the amine species with hydriodic acid (HI).

L-amines: hexylamine (Sigma Aldrich, 219703-100ML), decylamine (Sigma Aldrich, D2404-5G)

Acid: hydriodic acid (HI) (Sigma Aldrich, 752851-25G)

Abbreviations. For the ease of writing several abbreviations are used, which are summarized here: phenethylammonium (PEA), butylammonium (BA), hexylammonium (HA),

octylammonium (OA), decylammonium (DA), 4-fluoro-phenethylammonium (4FPEA), lead (Pb), iodide (I), methylammonium (MA), formamidinium (FA), two-dimensional (2D)

Synthesis. Layered perovskites, with the exception of $(HA)_2PbI_4$ and $(DA)_2PbI_4$, were synthesized under ambient laboratory conditions following the over-saturation techniques.[30–32] In a nutshell, the precursor salts LI, PbI_2, and AI were mixed in a stoichiometric ratio (2:1:0 for n = 1 and 2:2:1 for n = 2) and dissolved in γ-butyrolactone. The solution was heated to 70 °C and more γ-butyrolactone was added (while stirring) until all the precursors were completely dissolved. The resulting solutions were heated to 70°C and the solvent was left to evaporate. After 2-3 days, millimeter sized crystals formed in the solution, which was subsequently cooled down to room temperature. For this study, we drop cast some of the remaining supersaturated solution on a glass slide, heated it up to 50 °C with a hotplate and after the solvent was evaporated, crystals with crystal sizes of up to several hundred microns were formed. The saturated solution can be stored and re-used to produce freshly grown 2D perovskites within several minutes. We would like to note that drop cast n = 2 solutions form several crystals with different n values. However, n = 2 crystals can be easily isolated during the exfoliation (see next section) and the formation of n = 2 can be favored by preheating the substrate to 50°C before drop casting.

$(HA)_2PbI_4$ and $(DA)_2PbI_4$ were synthesized by dissolving PbI_2 (100 mg) in HI (800 µl) through heavy stirring and heating the solution to 90°C. After PbI_2 was completely dissolved a stoichiometric amount of the amine was added dropwise to the solution.

Exfoliation. The perovskite crystals of the thin film were mechanically exfoliated using the Scotch tape method (Nitto SPV 224). The exfoliation guarantees a freshly cleaved and atomically flat surface area for inspection, which is crucial to avoid emission from edge states and guarantee direct contact with the glass substrate. After several exfoliation steps, the crystals were transferred on a glass slide and were subsequently studied through the glass slide with a 100x oil immersion objective (Nikon CFI Plan Fluor, NA = 1.3). A big advantage of this technique is that the perovskites are encapsulated through the glass slide from one side and by the bulk of the crystal from the other side. It is important to use thick crystals to guarantee good self-encapsulation and prevent premature degradation of the perovskite flakes to affect the measurement.[32]

Exciton Diffusion Measurements. Exciton diffusion measurements were measured following the same procedure as Akselrod et al.[6,7] In short, a near diffraction limited exciton population was created using a 405 nm laser (PicoQuant LDH-D-C-405, PDL 800-D) and a 100x oil immersion objective (Nikon CFI Plan Fluor, NA = 1.3). Fluorescence of the exciton population was then imaged with a total 330x magnification onto an avalanche photodiode (APD, Micro Photon

Devices PDM) with a detector size of 20 µm. The laser and APD were synchronized using a timing board for time correlated single photon counting (Pico-Harp 300). The APD was capturing an effective area of around 60 x 60 nm (= 20 µm / 330). The APD was scanned through the middle of the exciton population in 60 or 120 nm steps, recording a time trace in every point. To minimize the degradation of the perovskites through laser irradiation, the perovskite flakes were scanned using an x-y-piezo stage (MCL Nano-BIOS 100), covering an area of 5 x 5 µm. Diffusion measurements were performed with a 40 MHz laser repetition rate and a laser fluence of 50 nJ cm^{-2} unless stated otherwise. The time binning of the measurement was set to 4 ps before software binning was applied.

For the temperature dependent measurements, the temperature was varied with a silicon heater mat (RS PRO, 245-499), using a PID temperature controller (Dwyer Instruments, Series 16C-3) connected to a type K thermocouple (Labfacility, Z2-K-1M) for feedback control. Here, a silicon heater mat was chosen over the Peltier element as a Peltier element expands during the heating process and causes mechanical vibrations that lead to drift.

X-ray diffraction (XRD). XRD was performed with a PANanaltical X'Pert PRO operating at 45 kV and 40 mA using a copper radiation source (λ = 1.5406 Å). The polycrystalline perovskite films were prepared by drop casting the saturated perovskite solutions on a silicon zero diffraction plate.

We use XRD measurements to extract the lattice spacing *d* of the inorganic layers of 2D perovskites by extracting the diffraction peak positions θ_n and using Bragg's law $n\lambda = 2d\cdot\sin(\theta_n)$. Supplementary Figures 1-2 show the XRD pattern of polycrystalline $(PEA)_2PbI_4$ and $(BA)_2PbI_4$ films, respectively. The extracted lattice spacings are consistent with previously reported values of these materials.[1,2] Supplementary Table 1 summarizes the XRD data of the perovskite crystals used in this study.

Supplementary References

1. Du, K. Z. *et al.* Two-Dimensional Lead(II) Halide-Based Hybrid Perovskites Templated by Acene Alkylamines: Crystal Structures, Optical Properties, and Piezoelectricity. *Inorg. Chem.* **56**, 9291–9302 (2017).
2. Billing, D. G. & Lemmerer, A. Synthesis, characterization and phase transitions in the inorganic–organic layered perovskite-type research papers. *Acta Crystallogr. Sect. B* **63**, 735–747 (2007).
3. Hu, J. *et al.* Synthetic control over orientational degeneracy of spacer cations enhances solar cell efficiency in two-dimensional perovskites. *Nat. Commun.* **10**, (2019).
4. Lemmerer, A. & Billing, D. G. Synthesis, characterization and phase transitions of the inorganic-organic layered perovskite-type hybrids $[(C_nH_{2n+1}NH_3)_2PbI_4]$, n = 7, 8, 9 and 10. *Dalt. Trans.* **41**, 1146–1157 (2012).
5. Paritmongkol, W. *et al.* Synthetic Variation and Structural Trends in Layered Two-Dimensional Alkylammonium Lead Halide Perovskites. *Chem. Mater.* **31**, 5592–5607 (2019).
6. Akselrod, G. M. *et al.* Subdiffusive Exciton Transport in Quantum Dot Solids. *Nano Lett.* **14**, 3556–3562 (2014).
7. Akselrod, G. M. *et al.* Visualization of exciton transport in ordered and disordered molecular solids. *Nat. Commun.* **5**, 3646 (2014).
8. Bouchaud, J.-P. & Georges, A. Anomalous diffusion in disordered media: Statistical mechanisms, models and physical applications. *Phys. Rep.* **195**, 127–293 (1990).
9. Papavassiliou, G. C. Three- and Low-Dimensional Inorganic Semiconductors. *Prog. Solid State Ch.* **25**, 125–270 (1997).
10. Leimkuhler, B. & Matthews, C. Rational Construction of Stochastic Numerical Methods for Molecular Sampling. *Appl. Math. Res. eXpress* (2012).
11. Risken, H. Fokker-Planck Equation. in *The Fokker-Planck Equation* 63–95 (Springer Series in Synergetics, 1984).
12. Mooney, J. & Kambhampati, P. Get the Basics Right: Jacobian Conversion of Wavelength and Energy Scales for Quantitative Analysis of Emission Spectra. *J. Phys. Chem. Lett.* **4**, 3316–3318 (2013).
13. Wright, A. D. *et al.* Electron-phonon coupling in hybrid lead halide perovskites. *Nat. Commun.* **7**, (2016).

14. Fu, M. *et al.* Unraveling exciton–phonon coupling in individual $FAPbI_3$ nanocrystals emitting near-infrared single photons. *Nat. Commun.* **9**, (2018).

15. Diab, H. *et al.* Narrow Linewidth Excitonic Emission in Organic-Inorganic Lead Iodide Perovskite Single Crystals. *J. Phys. Chem. Lett.* **7**, 5093–5100 (2016).

16. Fu, Y. *et al.* Incorporating Large A Cations into Lead Iodide Perovskite Cages: Relaxed Goldschmidt Tolerance Factor and Impact on Exciton− Phonon Interaction. *ACS Cent. Sci.* **5**, 1377–1386 (2019).

17. Gauthron, K. *et al.* Optical spectroscopy of two-dimensional layered $(C_6H_5C_2H_4NH_3)_2PbI_4$ perovskite. *Opt. Express* **18**, 5912 (2010).

18. Neutzner, S. *et al.* Exciton-polaron spectral structures in two-dimensional hybrid lead-halide perovskites. *Phys. Rev. Mater.* **2**, (2018).

19. Zhang, Y. *et al.* Optical Properties of Two-Dimensional Perovskite Films of $(C_6H_5C_2H_4NH_3)_2[PbI_4]$ and $(C_6H_5C_2H_4NH_3)_2(CH_3NH_3)[Pb_3I_{10}]$. *J. Phys. Chem. Lett.* **10**, 13–19 (2019).

20. Straus, D. B. *et al.* Longer Cations Increase Energetic Disorder in Excitonic 2D Hybrid Perovskites. *J. Phys. Chem. Lett.* **10**, 1198–1205 (2019).

21. Ni, L. *et al.* Real-Time Observation of Exciton-Phonon Coupling Dynamics in Self-Assembled Hybrid Perovskite Quantum Wells. *ACS Nano* **11**, 10834–10843 (2017).

22. Mauck, C. M. *et al.* Inorganic Cage Motion Dominates Excited-State Dynamics in 2D-Layered Perovskites $(C_xH_{2x+1}NH_3)_2PbI_4$ (x = 4–9). *J. Phys. Chem. C* **123**, 27904–27916 (2019).

23. Segall, V. Thermal Broadening of Exciton Levels in II-VI Compound Semiconductors. in *IX International Conference On The Physics Of Semiconductors, Moscow* 425–430 (1968).

24. Lee, J., Koteles, E. S. & Vassell, M. O. *Luminescence linewidths of excitons in GaAs quantum wells below 150 K. Physical Review B* **8**, (1986).

25. Rudin, S., Reinecke, T. L. & Segall, B. *Temperature-dependent exciton linewidths in semiconductors*. *Physical Review B* **42**, (1990).

26. Straus, D. B. *et al.* Direct Observation of Electron-Phonon Coupling and Slow Vibrational Relaxation in Organic-Inorganic Hybrid Perovskites. *J. Am. Chem. Soc.* **138**, 13798–13801 (2016).

27. Lee, J. H. *et al.* Resolving the Physical Origin of Octahedral Tilting in Halide Perovskites. *Chem. Mater.* **28**, 4259–4266 (2016).

28. Lee, J. H., Deng, Z., Bristowe, N. C., Bristowe, P. D. & Cheetham, A. K. The competition

between mechanical stability and charge carrier mobility in MA-based hybrid perovskites: insight from DFT. *J. Mater. Chem. C* **6**, 12252–12259 (2018).

29. Gélvez-Rueda, M. C. *et al.* Interconversion between Free Charges and Bound Excitons in 2D Hybrid Lead Halide Perovskites. *J. Phys. Chem. C* **121**, 26566–26574 (2017).

30. Yaffe, O. *et al.* Excitons in ultrathin organic-inorganic perovskite crystals. *Phys. Rev. B* **92**, 045414 (2015).

31. Ha, S. T., Shen, C., Zhang, J. & Xiong, Q. Laser cooling of organic-inorganic lead halide perovskites. *Nat. Photonics* **10**, 115–121 (2016).

32. Seitz, M., Gant, P., Castellanos-Gomez, A. & Prins, F. Long-Term Stabilization of Two-Dimensional Perovskites by Encapsulation with Hexagonal Boron Nitride. *Nanomaterials* **9**, 1120 (2019).
